# Reducing contacts to stop SARS-CoV-2 transmission during the second pandemic wave in Brussels, Belgium, August to November 2020

**DOI:** 10.2807/1560-7917.ES.2021.26.7.2100065

**Published:** 2021-02-18

**Authors:** Brecht Ingelbeen, Laurène Peckeu, Marie Laga, Ilona Hendrix, Inge Neven, Marianne AB van der Sande, Esther van Kleef

**Affiliations:** 1 Department of Public Health, Institute of Tropical Medicine, Antwerp, Belgium; 2 Department of Infectious Disease Prevention and Control, Common Community Commission, Brussels-Capital Region, Brussels, Belgium; 3 Julius Center for Health Sciences and Primary Care, Utrecht University, Utrecht, the Netherlands

**Keywords:** COVID-19, Coronavirus Infections, epidemiology, Europe, epidemiology, Pandemics, prevention and control, Pneumonia, Viral, epidemiology, Disease outbreak, Reproduction number, SARS-CoV-2

## Abstract

To evaluate the effect of physical distancing and school reopening in Brussels between August and November 2020, we monitored changes in the number of reported contacts per SARS-CoV-2 case and associated SARS-CoV-2 transmission. The second COVID-19 pandemic wave in Brussels was the result of increased social contact across all ages following school reopening. Physical distancing measures including closure of bars and restaurants, and limiting close contacts, while primary and secondary schools remained open, reduced social mixing and controlled SARS-CoV-2 transmission.

Belgium reported per capita the highest number of coronavirus disease (COVID-19)-related deaths and near highest number of cases worldwide and was heavily affected during Europe’s first and second pandemic wave, reporting a total of 21,634 deaths and more than 700,000 cases on 13 February 2021 [1]. We describe the effect of physical distancing and school reopening on the number of contacts reported by each confirmed case of severe acute respiratory syndrome coronavirus 2 (SARS-CoV-2) and on associated age-specific SARS-CoV-2 transmission patterns, using operational data from the COVID-19 contact tracing system of the Brussels region (Supplementary material) and case reports made available via the Belgian institute for health, Sciensano.

## Physical distancing measures in summer and autumn 

An increase in COVID-19 cases in July 2020 in Antwerp, Belgium’s second largest city, was reverted following a provincial ban on indoor events involving more than 100 people, a curfew, mandatory teleworking, mandatory wearing of face masks, and a national limit of five close contacts per household. Close contacts were individuals outside the household, with whom one could have contact for more than 15 min without keeping a distance of 1.5 m and not wearing a mask, excluding children younger than 12 years. However, soon after the end of the summer holidays, while case numbers were rising again, national and regional governments loosened physical distancing measures. Belgium’s Brussels-Capital region was first to observe a steep increase in cases but also to re-introduce physical distancing measures (Table). 

**Table:** Physical distancing measures and SARS-CoV-2 testing policy changes, Brussels region, Belgium, July–November 2020

Intervention	Start date
Cafés and restaurants may remain open until 1:00 and can take maximum 10 people per group	8 June
Sports allowed in groups of maximum 50 people	8 June
Maximum five close contacts^a^ per week	30 July
Reopening primary and secondary schools	1 Sep
Restart universities at 50–75% room occupancy, with face masks	14 and 21 Sep
Limit on number of close contacts suspended	30 Sep
Quarantine for high-risk contacts^b^ reduced from minimum 10 days to 7 days (if two negative tests)	30 Sep
Maximum three close contacts per week	6 Oct
Recommended teleworking	6 Oct
Bars and cafés closed at 23:00	6 Oct
Bars and cafés closed	8 Oct
Universities restrict seat occupancy to 20%	19 Oct
Testing restricted to symptomatic suspected SARS-CoV-2 cases (except for healthcare workers)	21 Oct
Quarantine for high-risk contacts extended to 10 days	21 Oct
Restaurants closed	26 Oct
Maximum one close contact outside the household per person and maximum four people in private gatherings (excluding < 12-year-olds)	26 Oct
Curfew between 10:00 and 18:00	26 Oct
Teleworking becomes the rule	26 Oct
Indoor sports prohibited (except < 12-year-olds)	26 Oct
Universities gradually switch to online learning	26 Oct
Maximum one close contact outside the household per household	2 Nov
Mandatory teleworking	2 Nov
Non-essential shops closed; professions involving physical contact or gatherings suspended	2 Nov
Extended autumn school holiday (31 Oct–15 Nov)	31 Oct

^a^ Close contacts are persons who are not part of your household, with whom contact can last for more than 15 min without keeping a distance of 1.5 m and not wearing a mask, excluding children younger than 12 years.

^b ^High-risk contacts are persons with whom the SARS-CoV-2 PCR-positive case had physical or cumulative 15 min non-physical contact within 1.5 m from 2 days before to 7 days after onset of symptoms.

Sources: https://www.commissioner.brussels/updates-covid-19; https://covid-19.sciensano.be/nl

## The second COVID-19 pandemic wave in Brussels region

**Figure 1:**
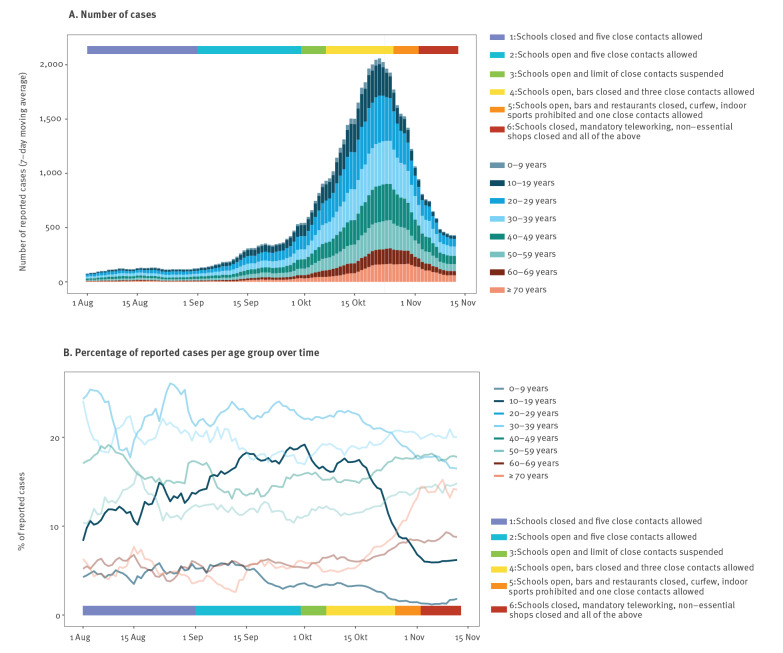
7-day moving average of SARS-CoV-2 confirmed cases reported, Brussels region, Belgium, 1 August–12 November 2020 (n = 415,412)

From 1 August to 12 November 2020, Brussels-Capital region reported 63,838 confirmed SARS-CoV-2 cases (5.2% of a population of over 1.2 million [2]), i.e. RT-PCR positive, among 415,412 SARS-CoV-2 PCR tests performed. The daily number of confirmed cases peaked on 20 October with 2,950 reported cases (Figure 1). SARS-CoV-2 test positivity was highest among 20–29-year-olds (7.4%, 13,436/181,940) and decreased with age to 4.3% positivity (4,913/114,637) among those 70 years and older (Supplementary Figure S1).

## Effect of physical distancing measures on the number of reported contacts of cases

**Figure 2:**
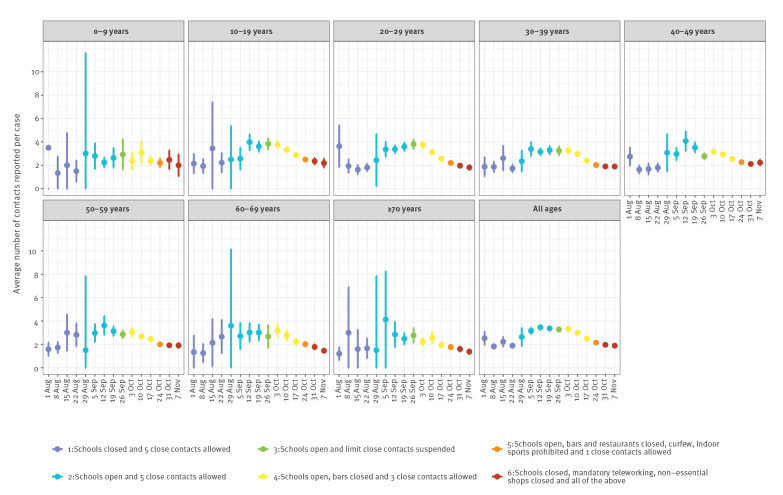
Weekly mean number of contacts reported per SARS-CoV-2 case (excluding cases not reporting any contacts), by age group, Brussels region, Belgium, 1 August–12 November 2020 (n = 24,166)

We compared differences in the mean weekly number of contacts reported per case to the telephone- and field agent-based contact tracing system, and confidence intervals (CI), assuming normality, at the start and end of each intervention period (Table). Following school reopening on 1 September, the mean number of reported contacts per case increased from 2.01 (95% CI: 1.73–2.29) in the last week of August to 2.83 (95% CI: 2.59–3.06) in the first week of September and further increased to 3.04 (95% CI: 2.93–3.15) by 30 September when the limit on the number of close contacts was suspended (Figure 2). A restriction to three close contacts and closure of bars on 6 and 8 October resulted in a gradual decrease in reported contacts per case from a mean of 2.81 (95% CI: 2.74–2.89) in the first week to 2.21 (95% CI: 2.16–2.25) 2 weeks later, just before contacts were further limited on 26 October. Following a limit to one close contact and closure of restaurants and sports facilities, the number of contacts per case further decreased to 1.94 (95% CI: 1.90–1.99), a 36.2% decrease compared with 30 September. When also shops were closed, teleworking became mandatory and schools started the autumn break, the mean number of reported contacts stagnated at 1.85 (95% CI: 1.78–1.91).

Weeks follow 7-day intervals from 1 August. Vertical bars indicate 95% confidence intervals. Colours indicate ongoing interventions or the intervention started that week. For trends in daily estimates and exact timing, see Supplementary Figure S2. For readability, the wide confidence intervals of the observation of the first week in the age group 0–9 years were removed. Of note, testing and related contact tracing for 0–6-year-olds was restricted to symptomatic cases only during the period of study.

The number of reported contacts per case was highest among 10–19-year-olds during our study period (3.11; 95% CI: 3.01–3.21); adults 70 years and older reported the lowest number (2.05; 95% CI: 1.93–2.18). However, over time, changes in the number of contacts following changes in physical distancing measures were largely similar across age groups, with the exception of the 0–9-year-olds (no changes observed) and adults 70 and older (less pronounced, Figure 2). Of note, testing and related contact tracing for 0–6-year-olds was restricted to symptomatic cases only during the period of study.

## Effect of the number of reported contacts per case on SARS-CoV-2 transmission

**Figure 3:**
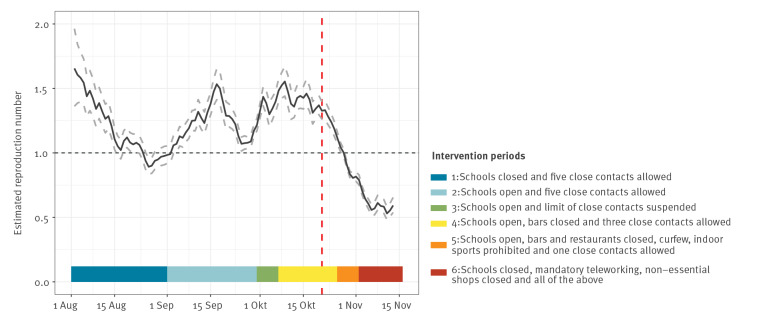
Estimated instantaneous reproduction number *R_t_* based on daily reported cases and a mean 5.2-day serial interval (95% credible interval: 4.4–6.0 [14]), Brussels region, Belgium, 1 August–12 November 2020 (n = 63,838)

We derived the instantaneous reproduction number *R_t_*_,_ i.e. the mean number of secondary cases that would arise from a primary case on a given day, from the daily number of reported cases, assuming an uncertain serial interval distribution (i.e. drawn from multiple truncated normal distributions with mean 5.19 days, 95% credible interval (CrI): 4.37–6.02), setting a 7-day sliding window, and estimating CrI using bootstrapping [3]. The *R_t_* peaked on 17 September at 1.48 (95% CrI: 1.35–1.63). Three weeks after the gradual restriction of close contacts (first three, then one) and the closure of bars, restaurants and sport facilities, *R_t_* had decreased by 44.6% to 0.82 (95% CrI: 0.79–0.85) (Figure 3). Even though a change in testing strategy to symptomatic cases only might have contributed to the drop in *R_t_*, the drop continued in the 2 weeks following the change. 

After 21 October (vertical dashed red line) asymptomatic contacts were excluded from SARS-CoV-2 testing. The dashed grey lines are 95% credible intervals. The horizontal dashed black line corresponds to a reproduction number of 1.

The analysis was done using the EpiEstim R package [3].

## Age-specific transmission patterns

**Figure 4:**
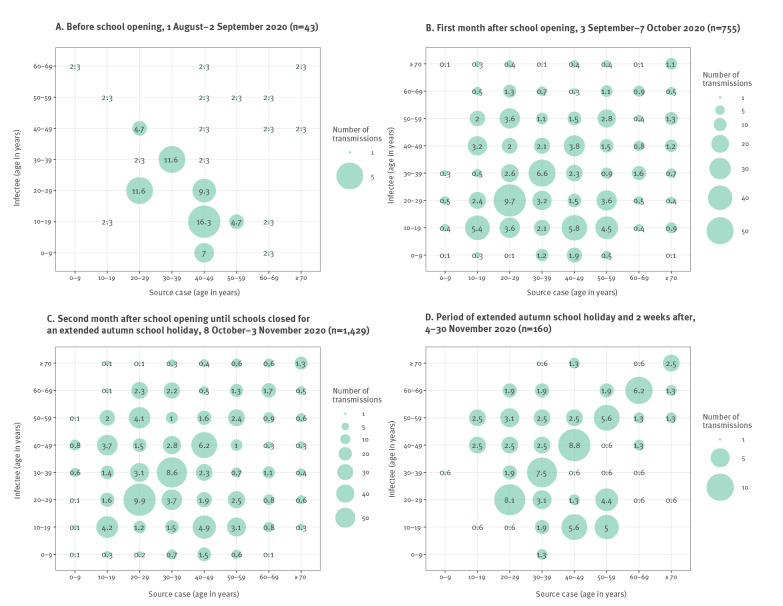
Transmission matrix between primary and secondary cases of all identified transmission events, Brussels region, Belgium, 1 August–30 November 2020 (n = 2,387)

Among 2,387 primary–secondary case pairs identified during the period 1 August to 30 November, transmission within the same age group was predominant (33.4%, 797/2,387). Infections originating from 10–19-year-olds were seldom recorded in August and November when schools were closed but testing of this group was low at these times as well (Figure 4A, Supplementary Figure S3). After schools reopened, transmission between all age groups became more apparent. In the month after reopening schools, 8.9% (67/755) of infections were from 10–19-year-olds to other age groups and 17.4% (131/755) from other age groups to 10–19-year-olds (Figure 4B). During extended autumn holidays and the closure of all non-essential services starting on 2 November (Figure 4D), intragenerational transmission was highest at 39.4% (63/160). Transmission within older age groups (≥ 50 years) became more frequent later in the second pandemic wave.

## Age-specific trends in reported SARS-CoV-2 cases

SARS-CoV-2 case reports among 10–19-year-olds increased throughout August and September (Figure 1B), coinciding with an increasing testing rate in this age group (spearman rank correlation coefficient = 0.74; p value < 0.001; Supplementary Figure S2). At the time schools reopened (1 September), we did not observe any significant change in the proportion of 10–19-year-olds among all diagnosed cases (adjusted for 4 days reporting delay; Poisson regression risk ratio 1.23; 95% CI: 0.79–1.94; Supplementary Figure S5). When asymptomatic contacts were excluded from SARS-CoV-2 testing (from 21 October onwards), the proportion of 10–19-year-olds fell from 16.9% of cases (3,478/20,535 during the 2 weeks preceding the change) to 9.9% (2,214/22,330 during the 2 weeks following the change, Figure 1B). The proportion of adults 70 years or older who tested positive increased from 5.2% (727/13,872) during the first 2 weeks of October to 13.8% (1,574/11,430) in the first 2 weeks of November (Figure 1B).

## Ethical statement

The study was approved by the Institutional Review Board of the Institute of Tropical Medicine (reference number 1423/20) and the Ethics committee of the Antwerp University Hospital (reference number 20/34/435).

## Discussion

September 2020 saw a persistent increase in SARS-CoV-2 cases in the Brussels region following increased social mixing across all ages, as inferred from trends in the number of reported contacts per case. Stringent physical distancing measures were introduced 1 month after a persistent increase in *R_t_*. These initial measures (a limit to three close contacts per person, a curfew, closure of bars and recommended teleworking) reduced reported contacts of cases by more than a third within 3 weeks, resulting in an *R_t_* < 1 by 29 October; they reduced social mixing sufficiently to control transmission, even with high case numbers and without closing schools or full lockdown.

In contrast to the first pandemic wave, primary and secondary schools remained open throughout the second wave. There is general consensus that children attending primary school contribute little to transmission [4]. In contrast, the role of teenagers and secondary schools is still much debated. Teenagers can transmit and show a viral load comparable to adults [5]. Nonetheless, several studies indicated either lower susceptibility or a higher proportion of asymptomatic individuals among teenagers which might result in fewer secondary infections originating from younger individuals [4,6-8]. Modelling studies investigating the role of secondary schools have shown that school closures can help alter transmission dynamics – albeit insufficiently for control and based on data from the first months of the pandemic with limited preventive measures in schools [e.g. 7,9,10]. Our findings confirm transmission among and from teenagers, with intergenerational transmission apparent following school opening. Nonetheless, their relative role was limited: transmission events from 10–19-year-olds to other age groups remained fewer than those from adults, and the proportion of cases among 10–19-year-olds did not significantly change after school reopening. After school reopening, the number of reported contacts per case increased across all age groups, suggesting a change in behaviour and mobility of all age groups, which may, at least in part, indirectly relate to school opening, and resulting in transmission particularly within the individual age groups, and an increased *R_t_*.

Epidemic growth among older adults was delayed when compared to that in younger age groups, similar to observations in other European countries. Transmission of SARS-CoV-2 varies between age groups and settings [11]. In a socially structured disease system, transmission of infectious agents among individuals with social networks less linked to the general population (e.g. nursing home residents) can increase disproportionately when a network-specific abundance threshold, which may be different from the conventional R > 1 for the spread of infections, is reached [12]. We hypothesise that this so-called percolation phenomenon may explain why transmission among older adults peaked later.

Our study had some limitations. Firstly, the number of reported contacts per case was smaller (mean in August: 2.0; 95% CI: 1.8–2.0) and less heterogenous than what participants in a Belgian contact survey reported (mean: 3.5 during the period 27 July to 10 August) [13]. This was probably a result of our analyses only considering high-risk contacts (physical or cumulative 15 min non-physical contact within 1.5 m) and a result of poor recall of context-specific accidental social contacts (e.g. public transport, bars) or reluctance to report contacts. Yet, age-specific differences were comparable, suggesting that the conclusions based on trends over time remain valid. Secondly, only a small proportion of cases were known contacts, indicating high volumes of undetected transmission or poor linking between data. Thirdly, a shift in testing policy to include only symptomatic cases from 21 October onwards is likely to have resulted in fewer identified transmission events involving children or teenagers because these groups more frequently present without or with mild symptoms. This shift in testing could have resulted in an underestimation of *R_t_* at the end of October. However, *R_t_* continued to decrease steadily after the change in testing strategy, suggesting that a true drop in transmission levels is likely. To determine a causal relationship between measures implemented and social mixing, in turn affecting SARS-CoV-2 transmission, we made sure we observed a strong correlation, coherence between the different analyses in the study, that no other change in policy or context could explain the effect observed, that the observed effect followed the introduction of a measure, and that there was a dose–response relationship such as between the number of contacts and *Rt*. Moreover, our findings are plausible, and are consistent with prior modelling and real-world studies on COVID-19 and other infectious agents.

## Conclusion

The second pandemic wave in Brussels was a result of increased social mixing across all ages in the absence of strict physical distancing measures. Limiting the number of close contacts per person and closure of bars and restaurants resulted in a rapid decrease in reported contacts of cases, sufficient to control SARS-CoV-2 transmission (lowering *R_t_* to < 1).

## Supplementary Data

699000 Supplement
