# Supplementary material for: Reducing contacts to stop SARS-CoV-2 transmission during the second pandemic wave in Brussels, Belgium, August to November 2020
Source: Euro Surveill. 2021 Feb 18;26(7):2100065. doi: 10.2807/1560-7917.ES.2021.26.7.2100065 (PMC7897911; doi:10.2807/1560-7917.ES.2021.26.7.2100065)
Supplement: Supplement [file 21-00065_Supplement_INGELBEEN.pdf]

Supplementary material

This supplementary material is hosted by Eurosurveillance as supporting information alongside the article *Reducing contacts to stop SARS-CoV-2 transmission during the second pandemic wave in Brussels, Belgium*, on behalf of the authors, who remain responsible for the accuracy and appropriateness of the content. The same standards for ethics, copyright, attributions and permissions as for the article apply. Supplements are not edited by Eurosurveillance and the journal is not responsible for the maintenance of any links or email addresses provided therein.

Processing of contact tracing and case report data

In May 2020, Belgium implemented a phone- and field agent-based contact tracing system. SARS-CoV-2 PCR-positive cases were phoned or visited, and asked to report who they had contact with, recording names and postal codes of high-risk contacts. High-risk (close) contacts, defined as persons with whom the SARS-CoV-2 PCR-positive case had physical or cumulative 15 minutes non-physical contact within 1.5m from 2 days before to 7 days after onset of symptoms of a confirmed SARS-CoV-2 case, were identified and recommended to undergo SARS-CoV-2 PCR testing, regardless of symptoms. For reported contacts aged 0-6 years, and from October 21 onwards across all ages, testing was restricted to symptomatic individuals only[1]. In primary schools, pupils and teachers in the same class of a confirmed case were considered low-risk contacts, therefore did not require testing, except if presenting symptoms. In secondary schools, the regular high-risk contact definition and testing criteria were applied.

To identify transmission events between primary (index) and secondary cases (contacts that tested SARS-CoV-2 positive within 3 weeks after the reported date of contact with the index case), we linked pseudonymised data on reported high-risk contacts generated by the contact tracing system with SARS-CoV-2 case data (including age) using a unique identifier based on first and last name. Homonyms that resulted in duplicates with the same unique identifier were excluded from the dataset.

We used 3 datasets from the Brussels region: (i) contacts reported by reported cases through contact tracing: date and index case ID; (ii) daily reported cases numbers; (iii) the primary-secondary case transmission events. We described case numbers, testing rates, test positivity, and mean numbers of high-risk contacts reported per SARS-CoV-2 case, by age group. We built a linear regression model to evaluate trends in the daily mean number of reported contacts by intervention period. We estimated the instantaneous reproduction number (Rt) from daily reported cases and analysed its correlation to the number of reported contacts. We described age patterns of identified transmission events in a matrix linking index and secondary cases. Data processing and analysis scripts are accessible on a GitHub repository: https://github.com/ingelbeen/covid19bxl.

A total of 52,484 cases were referred for contact tracing. Among these cases, 24,166 (46.0%) reported at least one contact, 61,754 in total. Matching operational case and contact databases resulted in a final 19,194 cases with recorded age and 51,177 contacts. The time between the last reported contact and contact tracing was median 2 days (interquartile range 0-5 days). Until 30 November, we traced back 2,443 reported contacts that tested SARS-CoV-2 positive within 3 weeks, yielding primary-secondary case pairs, 2,387 with age recorded.

Description of regression models

To visualise and describe changes in contact patterns over time, we fitted a segmented linear regression allowing for step and slope changes between distinct intervention periods as follows:

$$Y_t = \beta_0 + \beta_1 T_{t-2} + \beta_{2_i} X_t + \beta_{3_i} X_t T_{t-2} + \epsilon_t \quad (1)$$

Where Y_t is the expected mean number of contacts (individuals) on day *t*. T_t represents the day starting August 1, thus β_1 can be interpreted as the underlying trend in contact patterns without any changes in interventions. X_t represents a dummy variable indexing the 6 distinct intervention periods *i*, with β_2 and β_3 representing the step and slope change in contacts following the introduction of interventions. We added a 2-day lag for delay between the moment of the at risk contact with the case and reporting

[1] Sciensano. Classification of contacts for children. 10/08/2020. 2020 https://covid-19.sciensano.be/sites/default/files/Covid19/20200810_Advice_RAG_classification contacts children.pdf.

and identifying of that contact by the case, based on the median number of days between the last reported contact and when the concerned contact person was traced.

We describe changes in the proportion of daily reported cases ($I_{t_{10-19}}$) among teenagers in the months pre- and post-school opening (August to September), using Poisson regression with a log-link and offset term representing the total daily reported cases.

$$\log(I_{t_{10-19}}) \sim \log(I_{t_{total}}) + \beta_0 + \beta_2 T_{t-4} + \beta_3 X_t + \beta_4 X_t T_{t-4} + \beta_5 X_{test_{10-19}} + \beta_6 X_{weekend} \quad (2)$$

T_t represents the days from August until September, capturing the underlying trend pre-school opening, X_t represents a dummy variable indexing 0 and 1 before and after school opening respectively. We adjusted the periods for reporting delays by including a lag between exposure and case report (4 days). The daily number of tests performed among teenagers was accounted for and depicted by $X_{test_{10-19}}$ as well as whether the case was reported positive during the weekend $X_{weekend}$. We compared model fits using Akaike Information Criterion (AIC), assuming different time trends following school opening (i.e. no, vs a step vs a step and slope change). Models with and without adjustment for school provided similar fits (AICs of 364.9, 363.1, and 362.4 for a model with a step and slope change, a step change only and no change at all respectively). Of note, models with and without testing showed similar fits, while $X_{test_{10-19}}$ proved highly correlated with time.

Supplementary figures

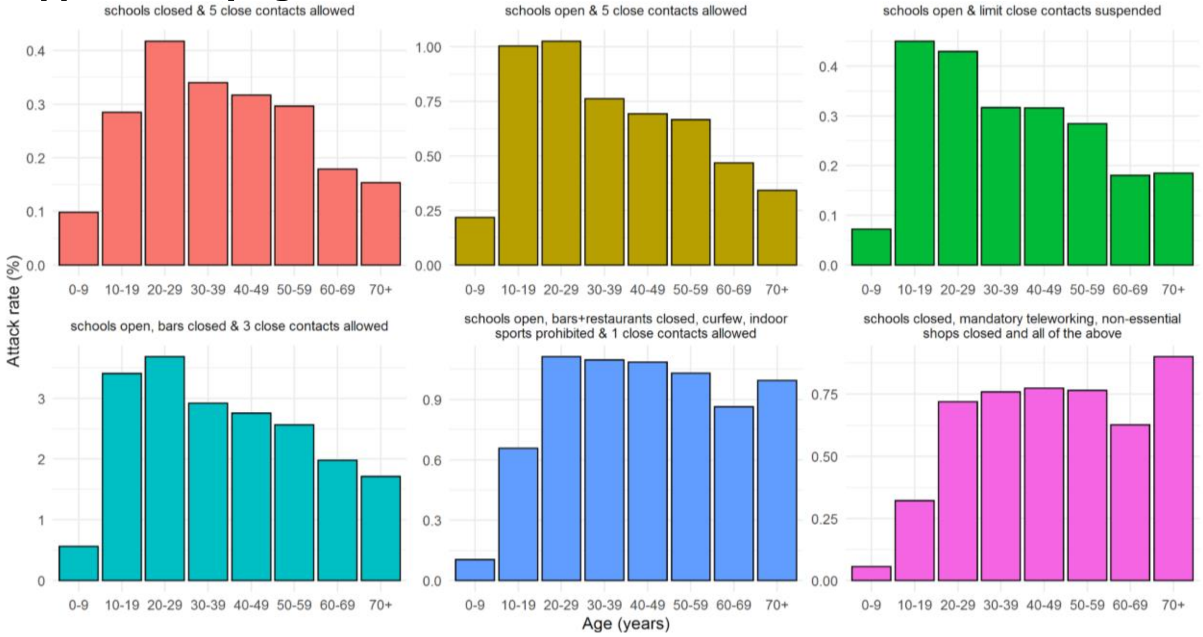

Supp Fig 1A. Percentage of the population which was SARS-CoV-2 confirmed by age group and by period of physical distancing measures. Source population numbers: https://statbel.fgov.be/en/themes/population/structure-population

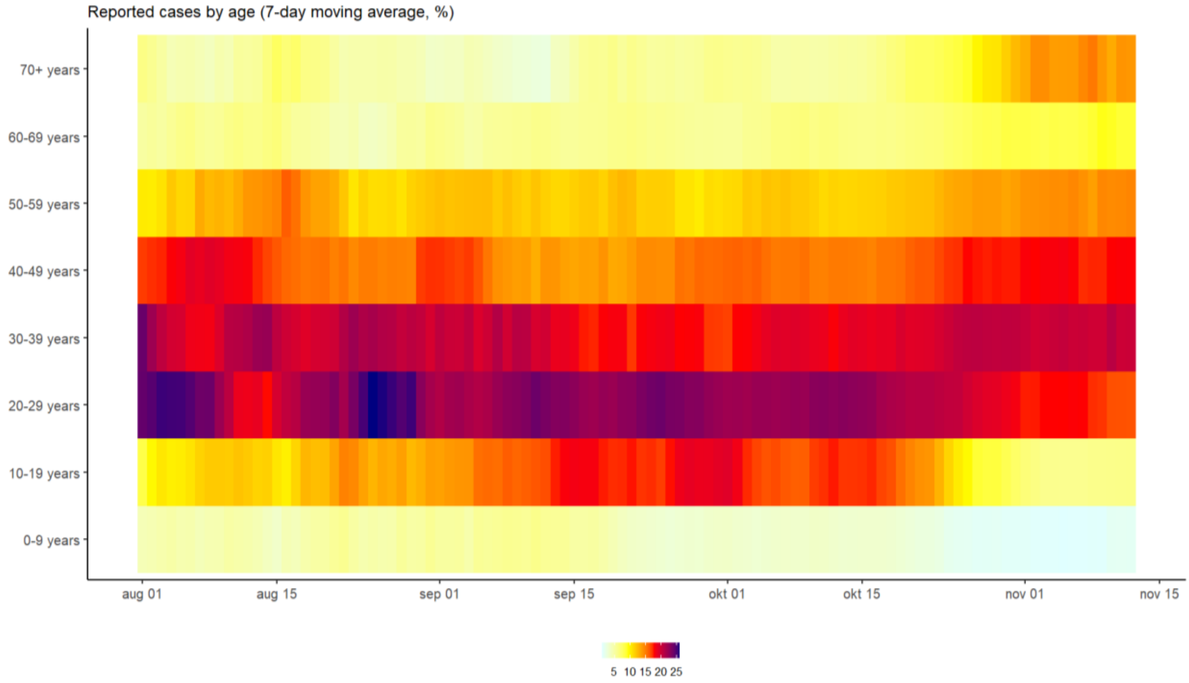

Supp Fig 1B. Percentage of the population which was SARS-CoV-2 confirmed by age group and by period of physical distancing measures. Source population numbers: https://statbel.fgov.be/en/themes/population/structure-population

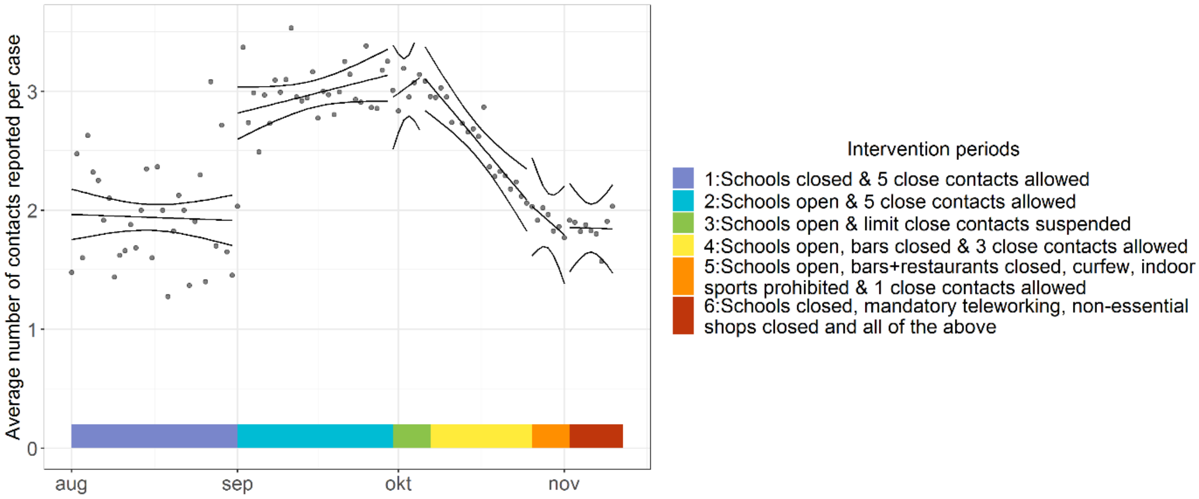

Supp Fig 2. Daily average number of contacts reported per SARS-CoV-2 case (excluding cases not reporting any contacts) with fitted estimated linear trends and 95% confidence intervals, using segmented linear regression with an interaction term for date and intervention periods, allowing for a step change. Lines are plotted as discontinuous for readability. The start of each segment in the linear regression is corrected for the median two days between the last reported contact and the interview.

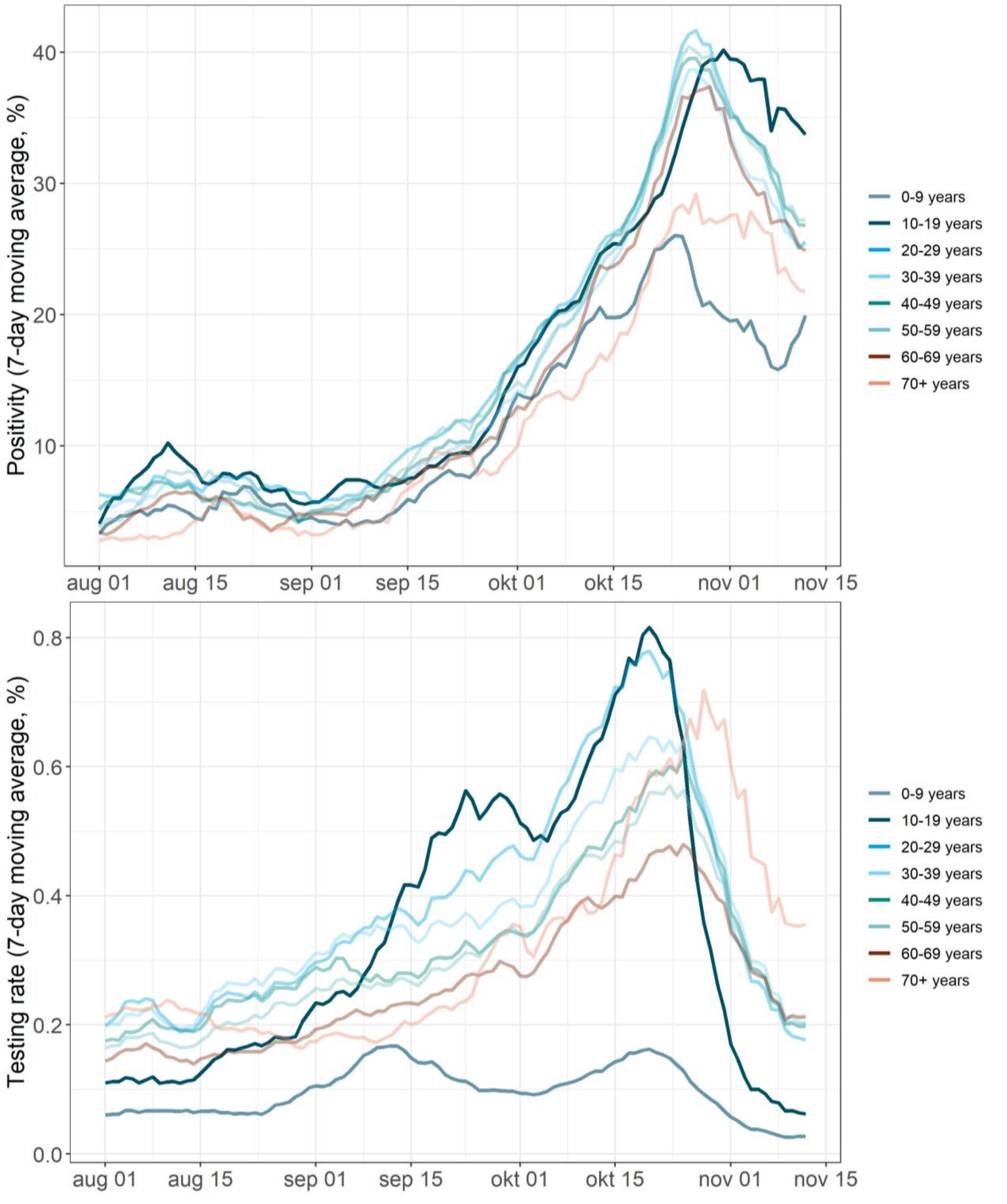

Supp Fig 3. Percentage of positive SARS-CoV-2 tests (A) and testing rate (B) by age group in the Brussels region. Source data: Sciensano and https://statbel.fgov.be/en/themes/population/structure-population

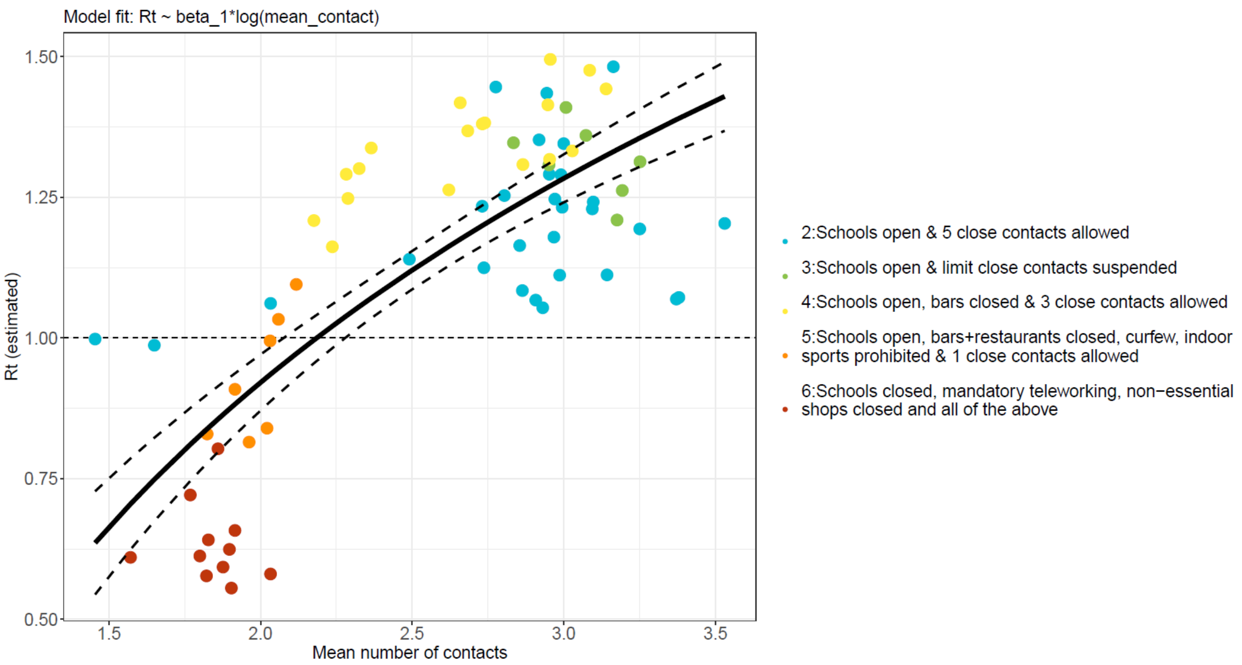

Supp Fig 4. Relationship between the number of reported contacts and reproduction number. Fitted linear regression model, regressing the instantaneous reproduction number (Rt) over the log daily mean number of contacts. August 2020 was excluded given the large variance we observed on the daily number of reported contacts.

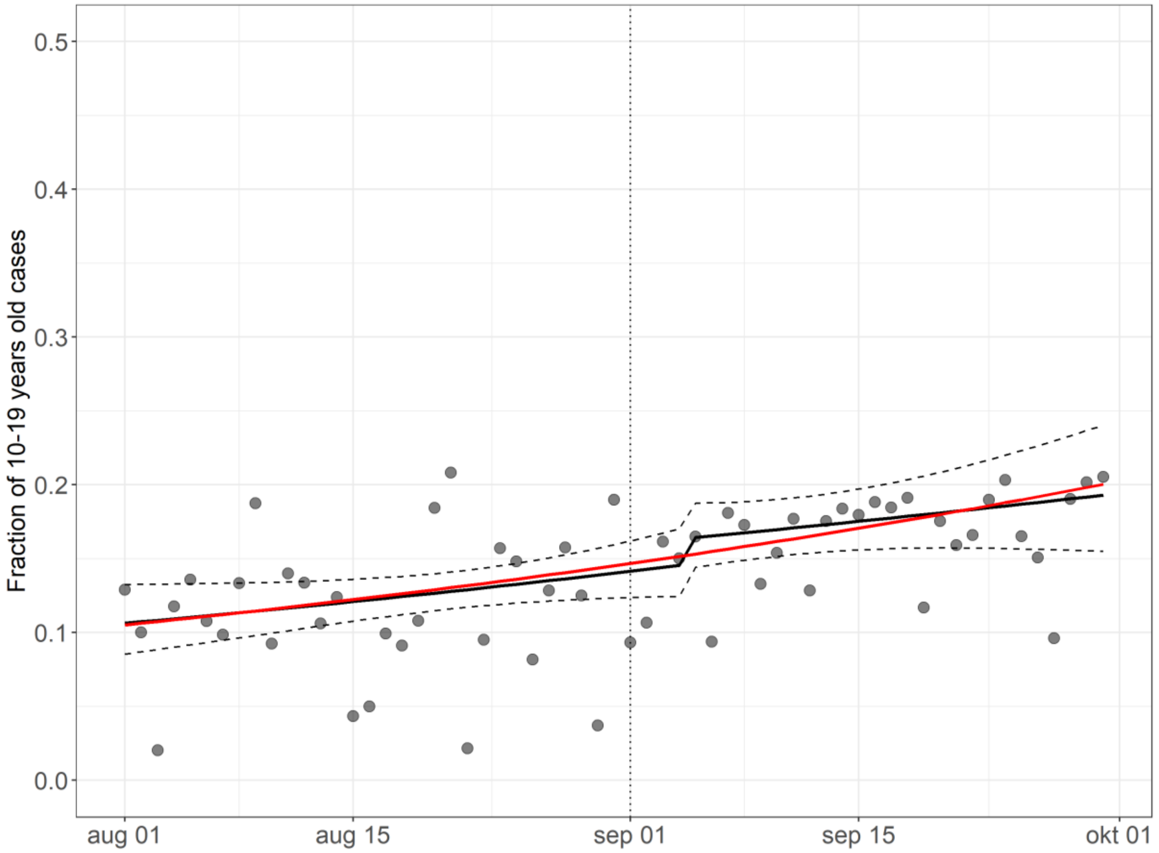

Supp Fig 5. Model fit of the fraction of 10-19 years olds among SARS-CoV-2 cases in Brussels before and after school opening, corrected for a 4-day test and report delay. Dotted line represents the timing of school opening. Red = model fit of a model assuming no step and slope change after school opening, setting variables representing weekend reporting to 0 (weekday) and number of tests among teenagers at its mean value. Black = model fit and 95% confidence interval of a model allowing for a step and slope change after school opening.

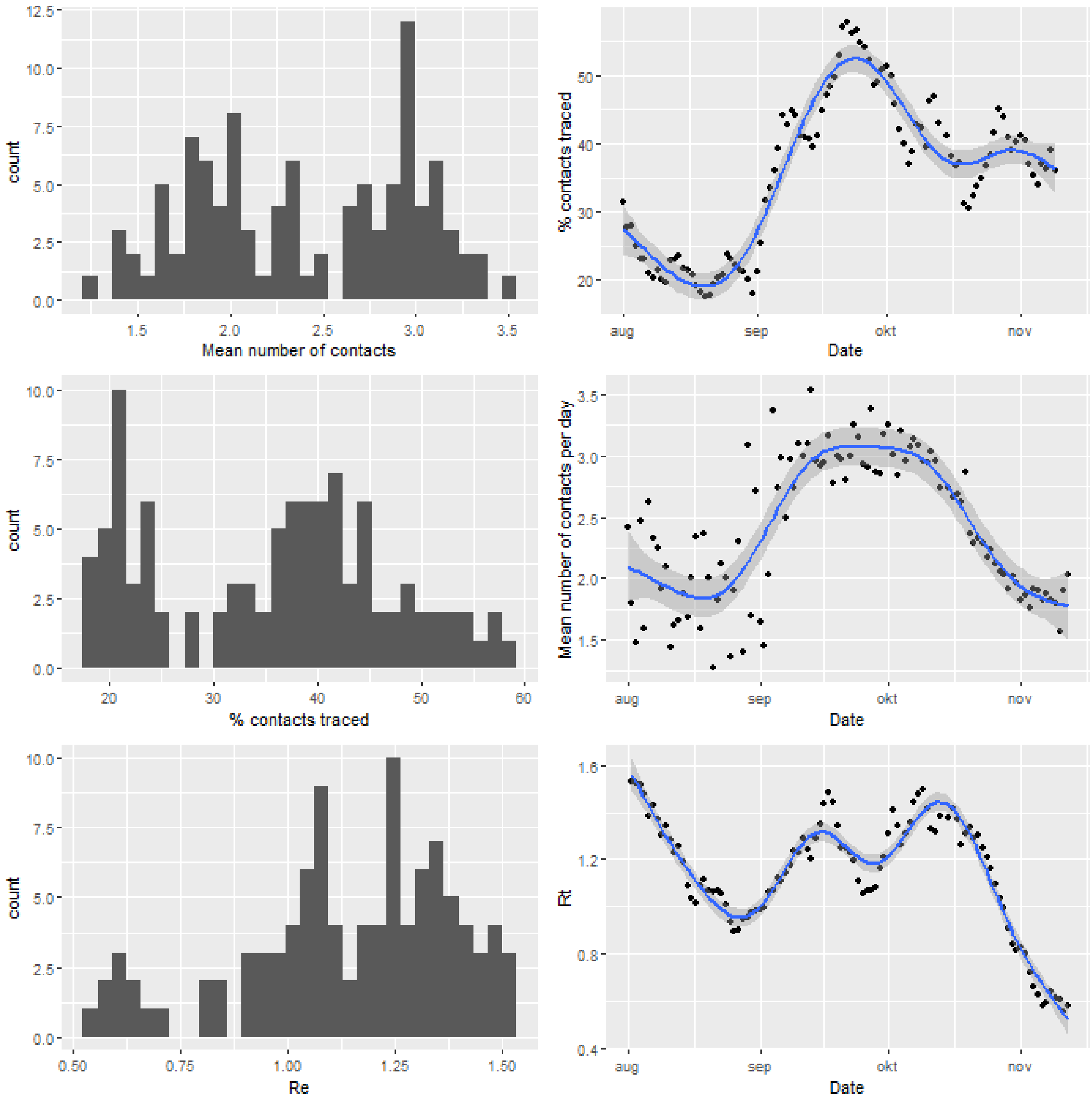

Supp Fig 6: Frequency distributions of the mean number of contacts reported, the percentage of reported contacts who could be successfully traced and the instantaneous reproduction number (Rt).
